# Assessing the Obesity Paradox in Alzheimer’s: A Systematic Review

**DOI:** 10.1002/alz.090083

**Published:** 2025-01-09

**Authors:** Daniela Delphus, Vaibhav Mittal, Prasanna Karur, Alexander Knight, Hadi Moussa, Deepesh Khanna

**Affiliations:** ^1^ Nova Southeastern Dr. Kiran C. Patel College of Osteopathic Medicine ‐ TBR, Clearwater, FL USA

## Abstract

**Background:**

Alzheimer’s Disease (AD) is a neurodegenerative disease that manifests as memory impairment, cognitive decline, and dementia. Of the preventive measures that exist for AD, research has been ongoing for modifiable risk factors, particularly obesity. The obesity paradox suggests that midlife obesity is a risk factor for AD, while later‐life obesity is a protective measure against AD. The purpose of this study is to examine the current literature on the obesity paradox for AD and determine its validity.

**Method:**

Articles were found on EMBASE using relevant keywords related to “Alzheimer’s,” “obesity,” “body mass index,” and “obesity paradox.” Manual de‐duplication was done and 2,784 studies were screened using a blind review method. Inclusion criteria were established for primary articles, human studies, and relevant to obesity paradox. Articles not published in English, reviews, and animal studies were excluded. Ultimately, 44 articles were selected for full‐text review.

**Result:**

Cerebrospinal fluid (CSF) biomarkers and brain structural changes were studied to evaluate the risk for AD in different patient groups. Multiple studies observed later life obese individuals had a decreased risk of AD. Compared to individuals with lower BMI, obese patients showed slower cognitive decline. Amyloid beta and tau protein are commonly associated with AD which several studies show vary among higher BMI individuals. These individuals showed lower loads of amyloid beta in the right hippocampus and decreased CSF tau protein. Higher BMI individuals showed increased total hippocampal and brain volume. A combination of these biomarker and structural changes shows the potential protective factor in obesity paradox.

**Conclusion:**

This review highlights the potential protective effect of later life obesity, suggesting that various biomarkers and structural changes among individuals may decrease the risk of AD. We highly recommend raising awareness and conducting further research to better understand this paradox.

